# Supplementary material for: Deep Brain Stimulation restores information processing in parkinsonian cortical networks
Source: medRxiv. 2024 Aug 28:2024.08.25.24310748. Preprint. [Version 1] doi: 10.1101/2024.08.25.24310748 (PMC11383511; doi:10.1101/2024.08.25.24310748)
Supplement: Supplement 1 [file media-1.pdf]

Supplementary Information for

Deep Brain Stimulation restores information processing in parkinsonian cortical networks

Charlotte Piette *et al*.

*Corresponding authors. Email: charlotte.piette@hotmail.fr , jtouboul@brandeis.edu , laurent.venance@college-de-france.fr

This file includes:

Figs. S1 to S7
Tables S1 to S6
Movies S1 to S2

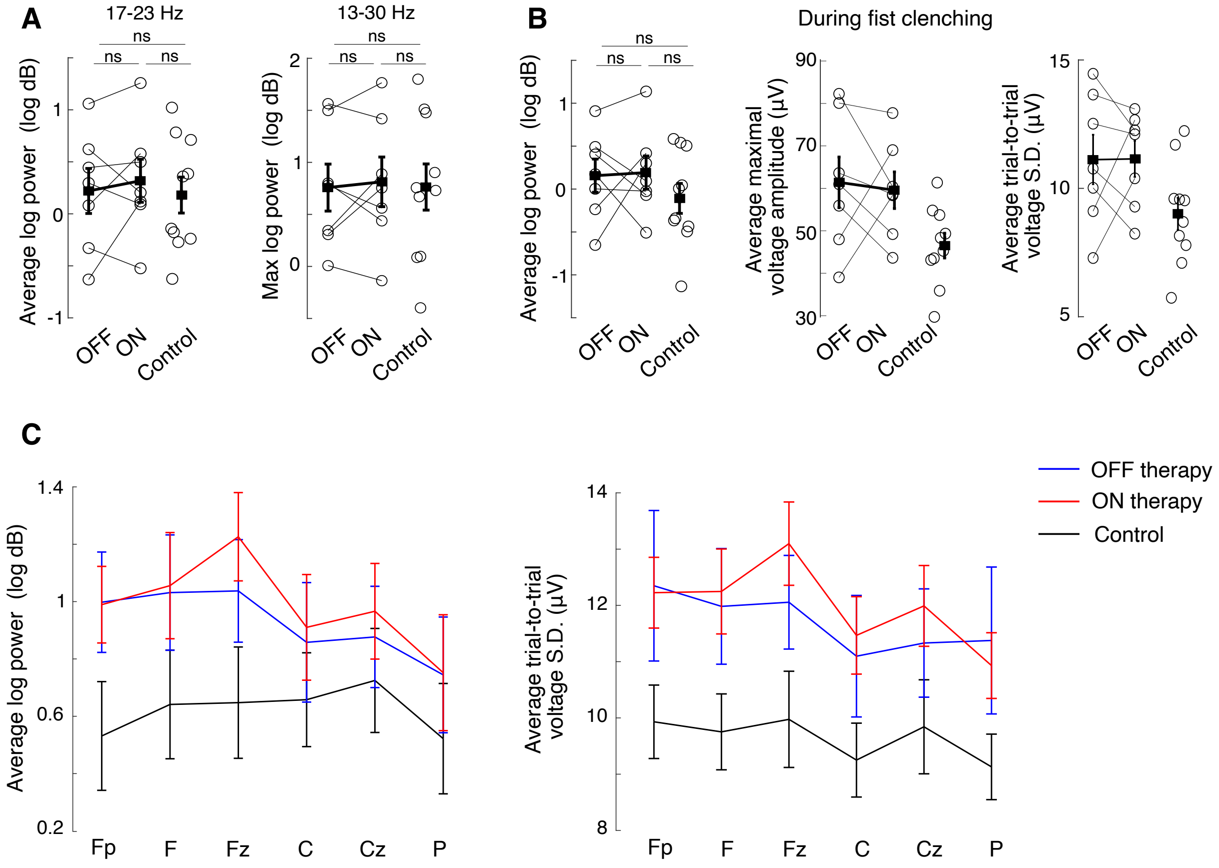

Fig. S1. EEG features did not differ between DBS OFF and ON conditions.

A. Alternative measures of beta band power across the entire finger tapping session, using the average log power over 17-23 Hz frequency range (left) or the maximal log power within the 13-30 Hz band (right) from the contralateral C electrode. No significant differences were observed between groups (paired Wilcoxon tests for OFF/ON condition: p=0.69 and p=0.69 respectively; independent Wilcoxon tests for OFF/Control: p=1 and p=0.96; for ON/Control: p=0.67 and p=0.96). **B.** EEG features measured from the contralateral C electrode during the initiation period of fist clenching show similar characteristics as for finger tapping. Left: Average beta-band power (13-30 Hz); Middle: Average maximal voltage amplitude; Right: Mean standard deviation of the voltage. Statistical tests: paired Wilcoxon tests for OFF/ON condition (p=0.94, p=0.69 and p=0.94); independent Wilcoxon tests for OFF/Control (p=0.42, p=0.55 and p=0.088) and for ON/Control (p=0.36, p=0.033 and p=0.043 respectively). **C.** The choice of analyzing the contralateral C electrode does not affect the observations previously made, as illustrated for the average beta-band power (left) and the mean standard deviation of the voltage (right).

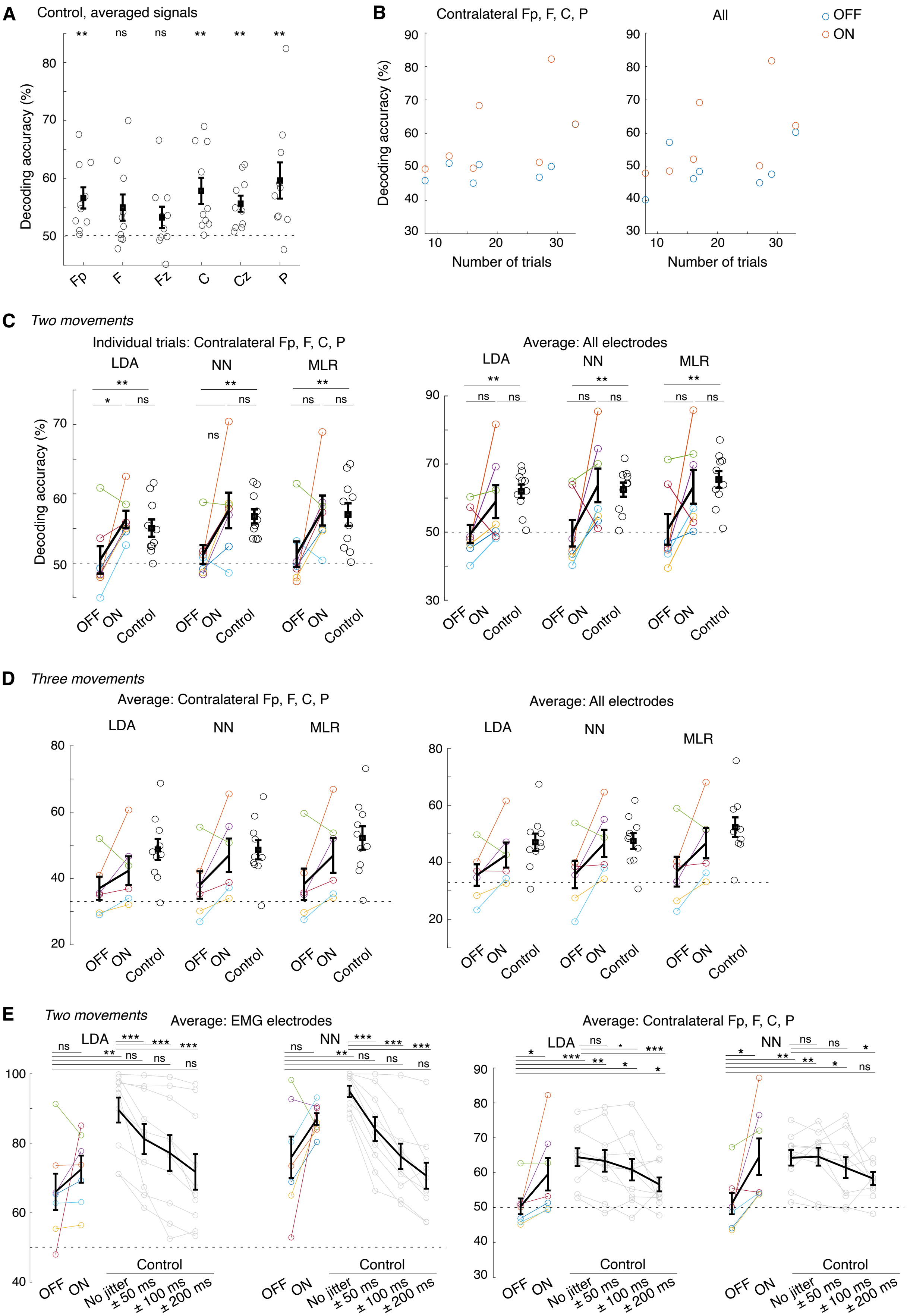

A
Control, averaged signals
Decoding accuracy (%)
Fp
F
Fz
C
Cz
P
B
Contralateral Fp, F, C, P
All
OFF
ON
Decoding accuracy (%)
Number of trials
C
Two movements
Individual trials: Contralateral Fp, F, C, P
Average: All electrodes
LDA
NN
MLR
Decoding accuracy (%)
OFF
ON
Control
D
Three movements
Average: Contralateral Fp, F, C, P
Average: All electrodes
LDA
NN
MLR
E
Two movements
Average: EMG electrodes
Average: Contralateral Fp, F, C, P
Control
No jitter
± 50 ms
± 100 ms
± 200 ms

Fig. S2. Altered decoding accuracy in movement identification from EEG signals in PD patients

A. Decoding accuracy for control subjects (each dot corresponds to one subject) using linear discriminant analysis on individual electrode signals. One-sample Wilcoxon test (chance level: 50%, **Table S2**). **B.** For each patient, the number of templates for training the classifiers could differ (but remained similar between ON/OFF therapy), such that we ensured that the absence of correlation between decoding accuracy and number of training templates. Pearson's correlation coefficient r and *p*-value when trained on (left) contralateral Fp, F, C, P electrodes and (right) all electrodes: OFF: $r = 0.60$, $p = 0.15$ / ON: $r = 0.54$, $p = 0.22$; OFF: $r = 0.40$; $p = 0.37$; ON: $r = 0.55$; $p = 0.20$, respectively. **C.** Decoding accuracy across three classifiers between patients ON and OFF therapy (paired Wilcoxon test) and with control subjects (independent Wilcoxon test). See **Table S3** for detailed statistics. Individual signals from four electrodes contralateral to the movement side (left), and averaged signals from all electrodes (middle) or from the contralateral C electrode (right) were used. **D.** Same as panel C but for classifying three movements, and for averaged signals from four electrodes contralateral to the movement (left) or from all electrodes (right). **E.** Decoding accuracy across two classifiers between patients ON and OFF therapy (paired Wilcoxon test) and with control subjects (independent Wilcoxon test and non-parametric Friedman test). Control data were artificially jittered (jitter drawn from a uniform distribution bounded at ± 50, 100 or 200 milliseconds). See **Table S4** for detailed statistics. Individual signals from the three EMG electrodes ipsilateral to the movement side (left) or from four electrodes contralateral to the movement (right).

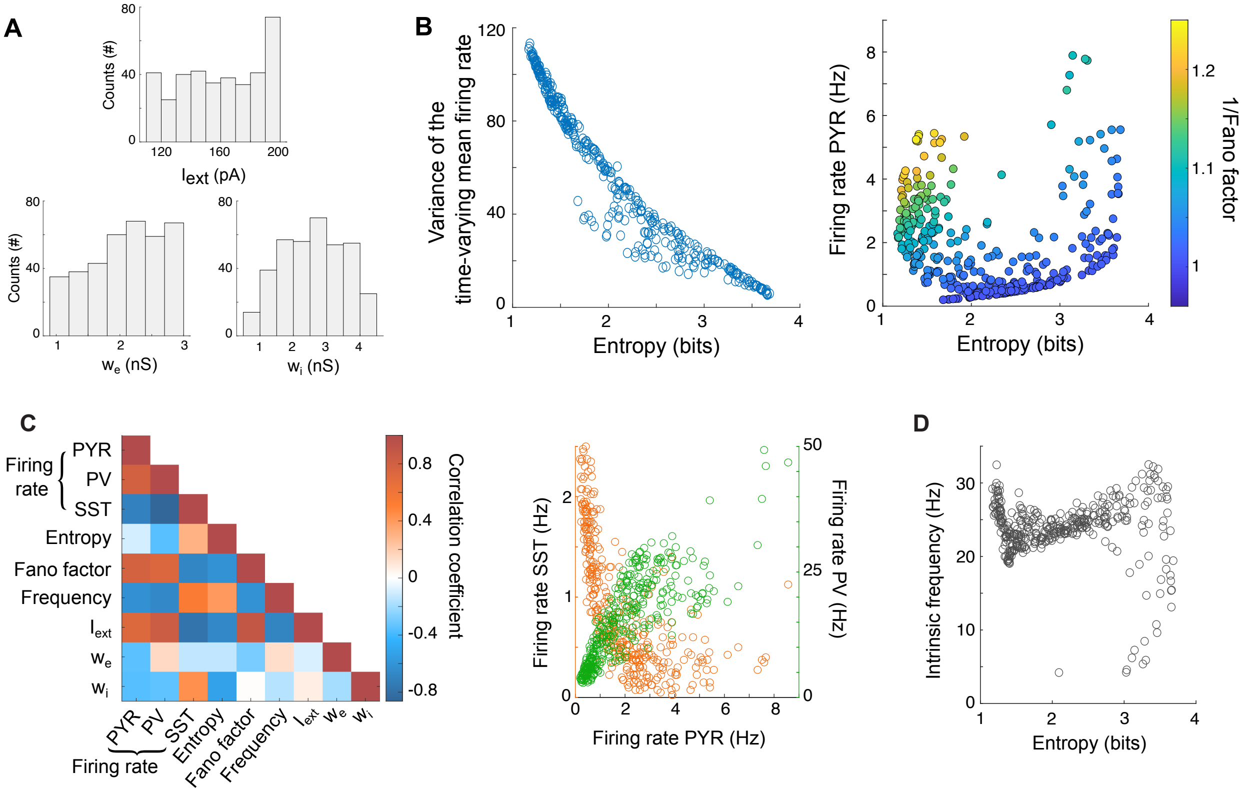

Fig. S3. Analysis of the activity of network spiking models in the OFF DBS condition
A. Distribution of the three variable parameters I_{ext}, w_e and w_i, after exclusion of trials (*Methods*). **B.** Alternative measures of synchronization: (left) correlation between the average variance of pyramidal spike times and entropy; (right) 1/Fano factor (color-coded) mapped onto the entropy and firing rate of pyramidal cells. **C.** Left: Pearson's correlation coefficients between model parameters and network activity. Right: Correlation between the firing rates of pyramidal cells and PV neurons (Spearman's r = 0.87, $p < 0.001$), and SST interneurons (Spearman's r = -0.82, $p < 0.001$). **D**. Sharpening of the intrinsic frequency to beta frequency range as the level of synchronization increases (when the entropy decreases).

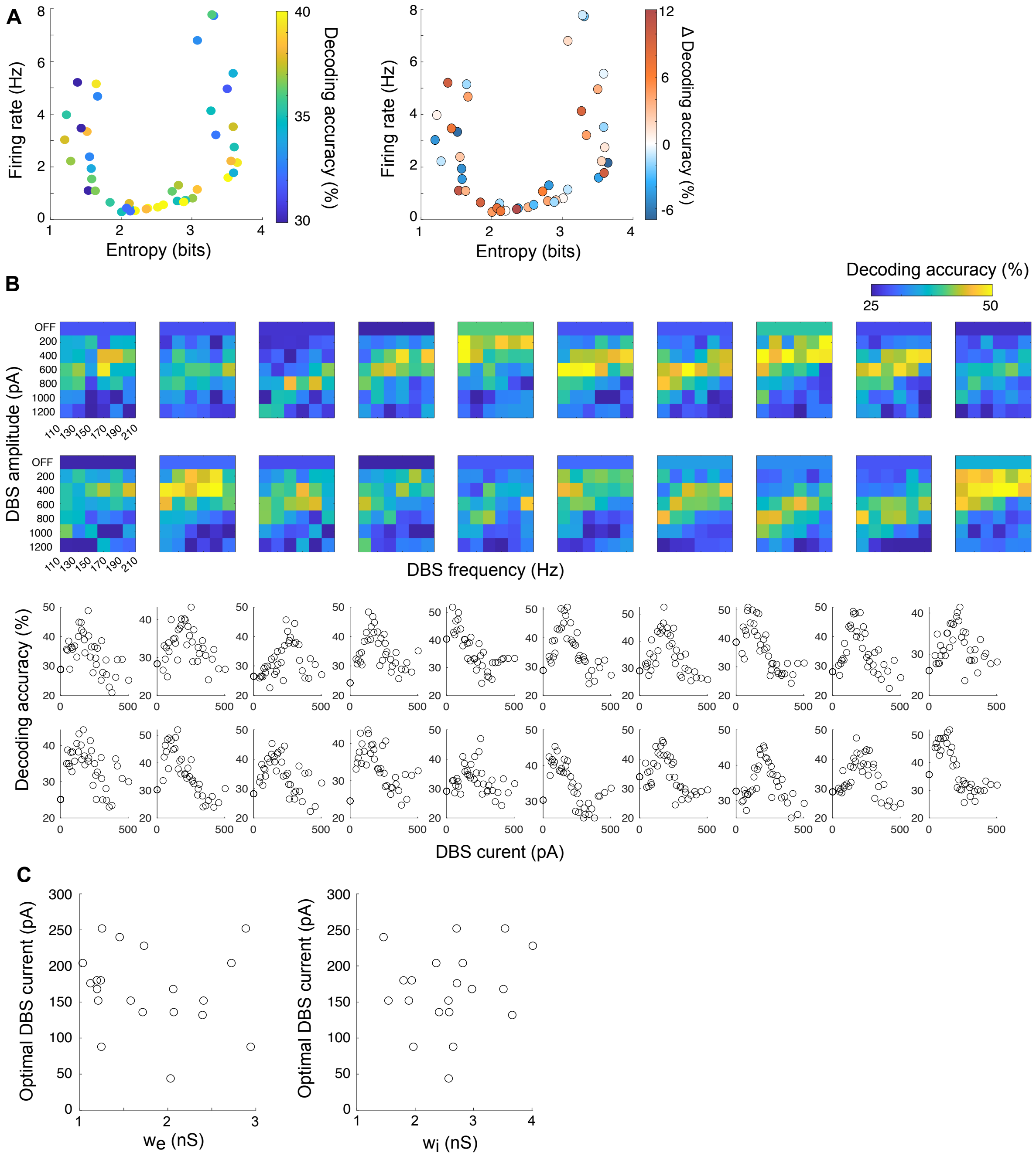

Fig. S4. Finding optimal DBS parameters with naturalistic stimuli

A. Left: Decoding accuracy when trained to recognize voices (3 voices, chance level: 33%) using multinomial logistic regression in the OFF DBS condition. Right: Changes in decoding accuracy under DBS (ON – OFF) when trained to recognize voices. Fixed DBS parameters (200 pA, 130 Hz) were used. **B.** Top: Decoding accuracy of the multinomial logistic regression when trained to recognize meaning (4 meanings, chance level: 25%) as a function of DBS frequency (x-axis) and amplitude (y-axis) for all models tested. Bottom: Projection of the decoding accuracy for all models on DBS current (x-axis), equal to DBS frequency x amplitude x pulse duration. **C**. Impact of network parameters w_e (left) and w_i (right) on the optimal DBS current (Pearson's correlation coefficient for w_e: r = -0.15, p = 0.53; for w_i: r = 0.16, p = 0.50).

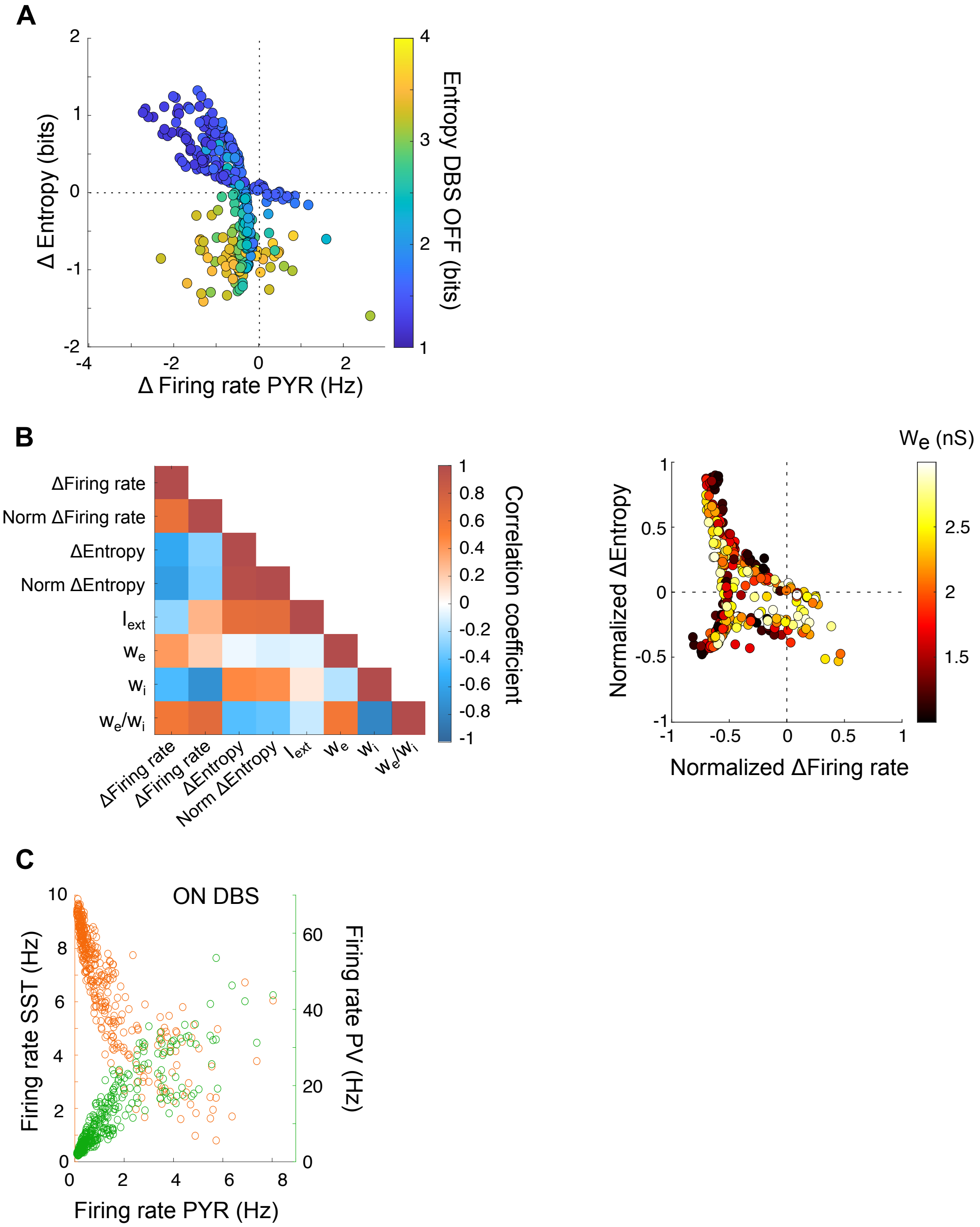

Fig. S5. Impact of DBS on the activity of network spiking models

A. Changes (absolute values) in entropy (y-axis) and firing rate (x-axis) between ON DBS and OFF DBS, as a function of the entropy OFF DBS (color-coded). **B.** Left: Correlation matrix between changes induced by DBS and network parameters (norm: normalized). High w_i was correlated with a decrease in firing rate (r = -0.44, $p < 0.001$) and an increase in entropy (r = 0.46, $p < 0.001$). High I_{ext} was especially correlated with an increase in entropy (r = 0.67, $p < 0.001$). Right: Impact of w_e on DBS-mediated changes. **C.** Correlation between the firing rate of pyramidal cells and that of interneurons (SST in orange, PV in green) under high-frequency DBS. The same correlation structure was observed in the OFF DBS condition, despite the strong recruitment of SST interneurons.

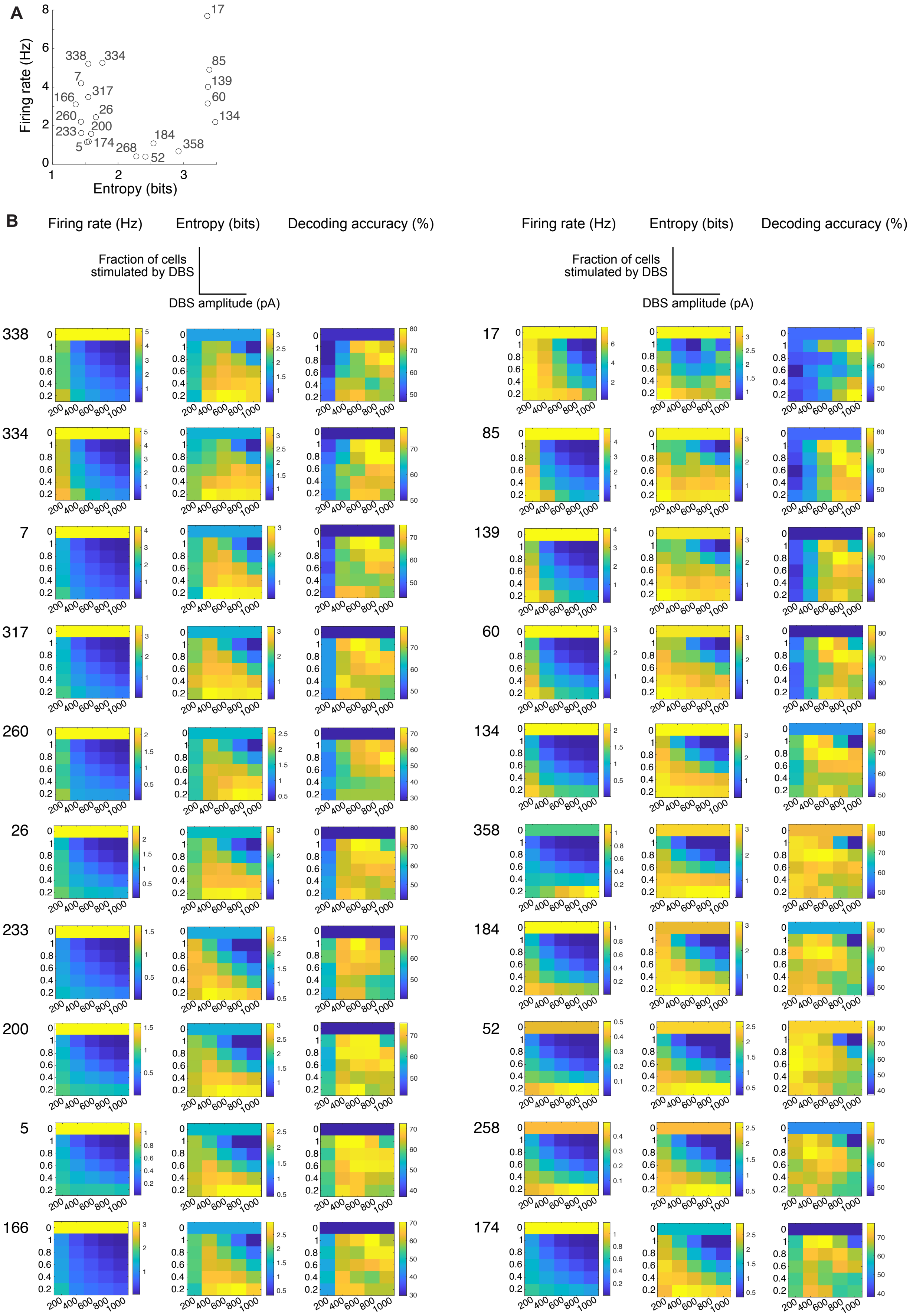
A
Firing rate (Hz)
Entropy (bits)
17
338
334
85
7
139
166
317
60
26
260
134
200
233
184
5
174
268
358
52
B
Firing rate (Hz)
Entropy (bits)
Decoding accuracy (%)
Fraction of cells stimulated by DBS
DBS amplitude (pA)
338
334
7
317
260
26
233
200
5
166
17
85
139
60
134
358
184
52
258
174

Fig. S6. Impact of varying the proportion of neurons recruited under DBS

A. Subset of network configuration in the firing rate/entropy diagram. Each network configuration is numbered. The 20 chosen configurations are scattered over the whole dynamic range observed from the set of 370 configurations. **B.** Evolution of the firing rate (Hz), entropy (bits) and decoding accuracy (LDA algorithm, %) as a function of the amplitude of DBS pulses (x-axis, DBS at 130 Hz) and the fraction of cells stimulated by DBS (y-axis) for each network configuration. Changes in firing rate, entropy and decoding accuracy under DBS are already markedly observed even when a small fraction of neurons is stimulated, without necessarily having to compensate with very high stimulation amplitude. For network configurations in which pyramidal cells show a high firing rate and DBS is less effective (eg. configurations 338, 334, 7, 17, 85, 139, 60), increasing DBS amplitude, even while maintaining a low fraction of stimulated cells, is particularly effective.

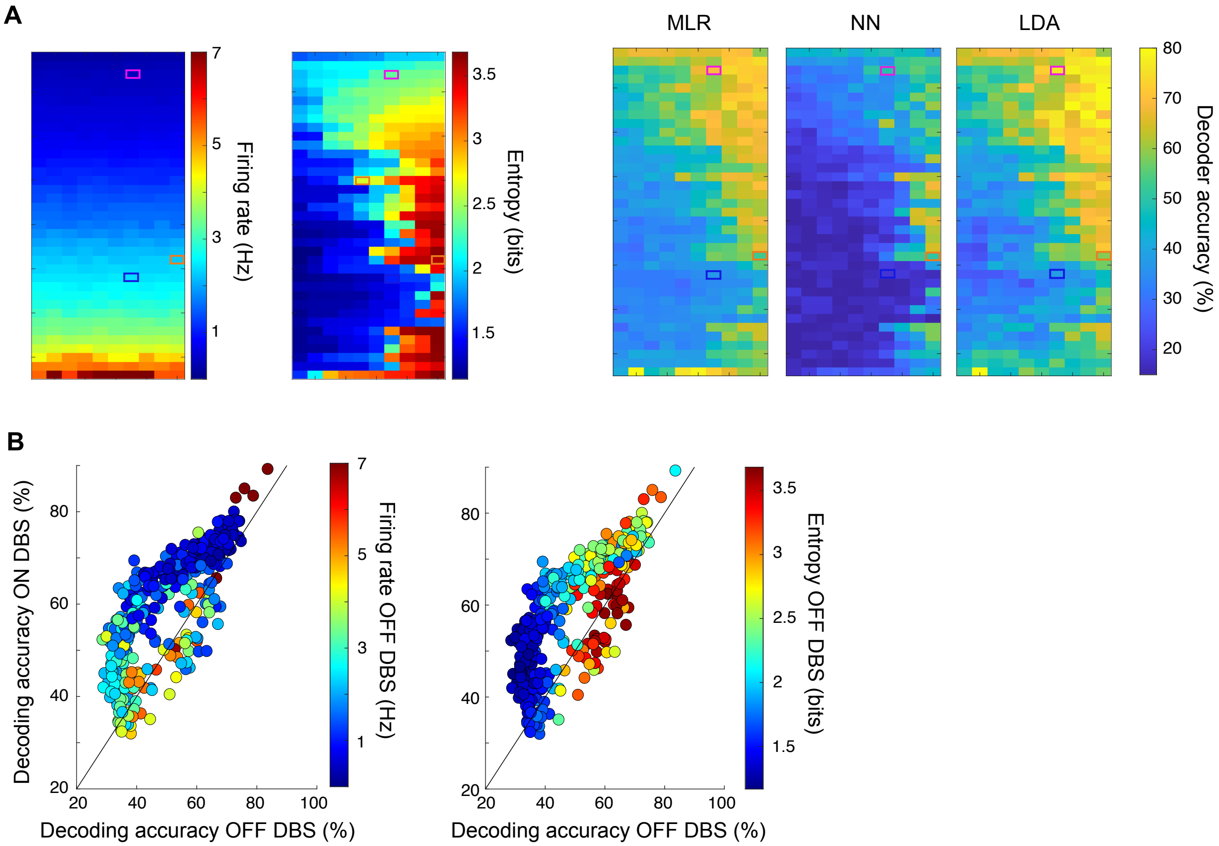

Fig. S7. Decoding accuracy on simple stimuli as a function of the network's activity regime

A. Network configurations ordered as a function of the firing rate and entropy (each square represents one network configuration). Decoding accuracy of the multinomial logistic regression (MLR), nearest-centroid (NN) and linear discriminant analysis (LDA) are compared using the same matrix arrangement. Overlaid colored rectangles refer to the three illustrative network profiles highlighted in **Fig. 2 & 4**. **B.** Comparison of MLR decoding accuracy OFF DBS (x-axis) and ON DBS (y-axis), with color-coded the firing rate OFF DBS (left) or the entropy OFF DBS (right).

	Sex	Age	Equivalence DOPA (mg)	Start of disease (years)	Side for movement execution
DBS-implanted parkinsonian patients	8 M, 2 F<br>Excluded: 3 M	57.4 ± 2.8 (max: 71, min: 45)	472 ± 98	7.1 ± 2.9	8 right<br>2 left
Control subjects	8 M, 2 F	57.7 ± 2.9 (max: 72, min: 45)	-	-	9 right<br>1 left

Table S1. Characteristics of the cohort for EEG recordings

Individual: 100 exemplars<br>Average: 20 exemplars	Mean ± SEM (n=10 control)	Wilcoxon sign-rank test p-value
**Figure 1E**		
Individual, Contra C	50.1 ± 0.5	0.5078
Average, Contra C	57.8 ± 2.3	0.002
Individual, Contra Fp, F, C, P	55.9 ± 2.4	0.0645
Individual, Ipsi Fp, F, C, P	52.2 ± 1.4	0.2324
Average, Contra Fp, F, C, P	60.8 ± 2.2	0.0059
Average, All	56.1 ± 2.3	0.0293
Permutation, All	58.5 ± 1.5	0.0059
**Figure S2**		
Permutation, Contra Fp	56.6 ± 1.8	0.0020
Permutation, Contra F	54.9 ± 2.3	0.0804
Permutation, Contra Fz	53.2 ± 1.9	0.1602
Permutation, Contra Cz	55.6 ± 1.4	0.0020
Permutation, Contra P	59.6 ± 3.1	0.0039

Table S2. Choice of electrode configuration for decoding movement

	Mean ± SEM<br><br>Per patient, training exemplars	**OFF therapy**<br><br>LDA<br>NN<br>MLR	**ON therapy**<br><br>LDA<br>NN<br>MLR	**Control**<br><br>LDA<br>NN<br>MLR	Paired Wilcoxon test OFF/ON<br>*p*-value	Wilcoxon test OFF/Control & ON/Control<br>*p*-value
2 movements	Average, Contra Fp, F, C, P	50.4 ± 2.3<br>51.1 ± 3.1<br>51.1 ± 4.0	59.5 ± 4.7<br>64.6 ± 5.2<br>63.8 ± 5.4	64.4 ± 2.6<br>64.3 ± 2.2<br>66.6 ± 2.8	0.0156<br>0.0312<br>0.0781	<0.001 and 0.1932<br>0.0068 and 0.9623<br>0.0097 and 0.6009
	Individual, Contra Fp, F, C, P	50.4 ± 2.0<br>51.1 ± 1.4<br>51.3 ± 1.8	56.3 ± 1.2<br>57.6 ± 2.6<br>57.6 ± 2.2	55.1 ± 1.3<br>56.7 ± 1.0<br>57.0 ± 1.6	0.0469<br>0.0781<br>0.0781	0.0431 and 0.3148<br>0.0046 and 0.8868<br>0.0025 and 0.9623
	Average, All	49.4 ± 2.7<br>49.7 ± 3.9<br>50.8 ± 4.5	59.0 ± 4.8<br>63.8 ± 4.9<br>63.3 ± 5.0	62.0 ± 1.9<br>62.5 ± 2.1<br>65.5 ± 2.5	0.1562<br>0.1094<br>0.0781	0.002 and 0.3638<br>0.0136 and 0.9623<br>0.033 and 0.6009
	Average, Contra C	50.4 ± 2.6<br>51.1 ± 3.5<br>51.0 ± 3.3	56.5 ± 3.4<br>60.8 ± 4.4<br>59.2 ± 3.9	64.4 ± 2.6<br>64.3 ± 2.2<br>66.6 ± 2.8	0.2188<br>0.2188<br>0.2969	0.0031 and 0.0553<br>0.0185 and 0.4173<br>0.0046 and 0.1932
3 movements*	Average, Contra Fp, F, C, P	37.1 ± 3.7<br>38.0 ± 4.8<br>38.2 ± 5.2	42.4 ± 4.3<br>47.0 ± 5.0<br>46.9 ± 5.2	48.8 ± 3.2<br>48.6 ± 2.9<br>52.2 ± 3.5	0.2188<br>0.1562<br>0.1562	0.0420 and 0.2198<br>0.0420 and 0.7925<br>0.0420 and 0.4923
	Average, All	35.5 ± 3.7<br>35.7 ± 4.8<br>36.7 ± 5.2	42.6 ± 4.4<br>46.6 ± 4.8<br>46.7 ± 5.3	47.0 ± 3.0<br>47.5 ± 2.8<br>52.3 ± 3.4	0.2188<br>0.0938<br>0.1562	0.0312 and 0.3132<br>0.0420 and 0.7128<br>0.0312 and 0.4278

*For two movements: n=7 OFF/ON therapy; for three movements: n=6 OFF/ON therapy.
In control patients, n=10.

Table S3. Summary of movement decoding accuracy from EEG signals

Mean ± SEM<br><br>Per patient, training exemplars	**OFF therapy**<br><br>LDA<br>NN	**ON therapy**<br><br>LDA<br>NN	**Control**<br>LDA<br>NN				Paired Wilcoxon test OFF/ON p-value	Non-parametric Friedman test (Control)	Wilcoxon test OFF/Control p-value			
			No jitter	± 50 ms	± 100 ms	± 200 ms						
Average, EMG electrodes	66.0 ± 5.2<br>75.9 ± 6.0	72.5 ± 3.9<br>87.0 ± 1.7	89.6 ± 3.6<br>94.9 ± 1.6	81.2 ± 4.3<br>84.1 ± 3.5	77.2 ± 5.2<br>76.2 ± 3.6	71.8 ± 5.1<br>70.6 ± 3.7	0.156<br>0.219	$\chi^2 = 27.7$<br>$p<0.001$<br><br>$\chi^2 = 27.7$<br>$p<0.001$	0.00308<br>0.00967	0.0553<br>0.315	0.193<br>0.475	0.601<br>0.219
Average, Contra Fp, F, C, P	50.4 ± 2.3<br>51.1 ± 3.1	59.5 ± 4.7<br>64.6 ± 5.2	64.4 ± 2.6<br>64.3 ± 2.2	63.4 ± 3.1<br>64.6 ± 2.6	60.8 ± 3.1<br>61.4 ± 3.1	56.6 ± 2.0<br>58.3 ± 1.9	0.0156<br>0.0313	$\chi^2 = 10.2$<br>$p=0.017$<br><br>$\chi^2 = 6.96$<br>$p=0.073$	<0.001<br>0.00679	0.00967<br>0.00967	0.0136<br>0.0330	0.0136<br>0.0553

Table S4. Comparison of movement decoding accuracy from EMG and EEG signals in presence of jitter

	Pyramidal cells	PV cells	SST cells
g_{leak} (nS)	6	5	5
C (pF)	180	80	80
V_{thres} (mV)	-49	-52	-53
Δ_{thres} (mV)	1	1	5
a (nS)	4	0	4
b (pA)	100	0	90
τ_w (ms)	100	15	40
σ	5	5	5
I_{ext}(pA)	variable	50	25

Table S5. Intrinsic parameters of the model for each neuronal population

p, w	Pyramidal cells	PV cells	SST cells
Pyramidal cells	p = 0.5, w =0.5 nS	p = 0.4, variable w_e	-
PV cells	p =0.4, variable w_i	p = 0.6, w= 2.5 nS	p = 0.1, w = 1.6 nS
SST cells	p = 0.4, w = 2.2 nS	p = 0.3, w = 2.4 nS	p= 0.1, w = 1.6 nS

Table S6. Connectivity parameters between neuronal populations

Movie S1.
Example of movement execution from a control patient

Movie S2.
Example of movement execution from a parkinsonian patient in OFF and ON therapy
